# Functional antigen matching in corneal transplantation: matching for the HLA-A, -B and -DRB1 antigens (FANCY) – study protocol

**DOI:** 10.1186/1471-2415-14-156

**Published:** 2014-12-13

**Authors:** Daniel Böhringer, Gabriele Ihorst, Birgit Grotejohann, Julia Maurer, Eric Spierings, Thomas Reinhard, on behalf of the FANCY study group

**Affiliations:** Eye Center, Medical Center, University of Freiburg, Freiburg, Germany; Clinical Trials Unit, Medical Center, University of Freiburg, Freiburg, Germany; Laboratory for Translational Immunology, University Medical Center Utrecht, Utrecht, The Netherlands

**Keywords:** Penetrating keratoplasty, HLA matching, graft rejection, HLAMatchmaker, minor transplantation antigens, operational tolerance

## Abstract

**Background:**

Penetrating keratoplasty can commonly restore vision in corneal blindness. However, immunological graft reactions may induce irreversible graft failure in a substantial percentage. Repeat keratoplasties in turn are associated with increased risk of graft failure and possibly irreversible blindness. Topical as well as systemic immunosuppressants are administered for prophylaxis. However, severe adverse effects limit long-term usage. By contrast, matching for transplantation antigens might be effective for a long time.

**Methods:**

FANCY is a prospective, controlled, randomised, double-blind, multi-centre clinical trial with two parallel arms. The primary objective is to evaluate superiority of the proposed HLA matching strategy in comparison to random graft assignment with respect to the primary endpoint ‘time to first endothelial graft rejection’. Relevant inclusion criteria are age over 18 years and waiting for penetrating or endothelial lamellar keratoplasty. The most important exclusion criteria are abuse of medication and/or drugs and an anticipated waiting time for an HLA match longer than 6 months. After randomisation, patients either receive a HLA-matched graft (experimental intervention) or a random graft (control intervention). The calculated sample size is 620 patients. The trial started in 2009 with a recruitment period of 24 months. A total of 654 patients were included during this time.

**Discussion:**

The primary goal of FANCY is to assess whether histocompatibility matching is feasible and effective in the broad clinical routine. However, during the course of the trial, the landscape of keratoplasty changed due to the rise of Descemet Membrane Endothelial Keratoplasty (DMEK). Nowadays, immune reactions are confined mostly to the ‘high-risk’ subgroups. If we would design FANCY in 2014, we would narrow down the inclusion criteria to include only the high risk patients and accept longer waiting times for a matching donor here.

**Trial registration:**

The unique identifying number of the FANCY trial is NCT00810472.

## Background

### Graft rejection after keratoplasty

Corneal diseases are among the five most common causes of blindness. FANCY was designed back in 2007. At that time, penetrating keratoplasty was the undisputed gold standard for corneal transplantation. This procedure can restore vision in the majority of affected patients due to the ocular immune privilege. Nevertheless, immunological graft reactions are a major barrier towards long-term success. They give either rise to irreversible graft failure immediately or at least promote late graft failure from endothelial cell loss. Penetrating repeat keratoplasties due to immunological graft failures are considered at high immunological risk irrespective of primary indication. This predicament commonly initiates a vicious cycle in affected patients due to increased risk of rejections in consecutive repeat keratoplasties.

### Current strategies of secondary prophylaxis

Immune reactions cannot currently be sufficiently prevented in the postoperative course of penetrating keratoplasty: on average, 18% in normal-risk penetrating keratoplasty and 75% of high-risk cases are affected - despite standard medical prophylaxis from topical steroids and in most high-risk situations additional systemic immunosuppression. These regimens have to be discontinued at some point in time due to the accumulating risk of severe adverse drug reactions: topical steroids commonly induce complications such as cataract or glaucoma. Systemic immunosuppressants like Cyclosporine A or Mycophenolate Mofetil may be effective from pilot studies [1] but have the potential to induce kidney failure as well as malignancies in the long run. Furthermore, this approach is hampered by the off-label mode of prescription and malcompliance.

### Considerations on primary prophylaxis

We are aware of no data on the molecular mechanisms of allorecognition of the corneal graft in the human situation. However, autologous grafts (such as in auto rotational keratoplasty) are never rejected. Both in-vitro and in-vivo data point towards a central role of the HLA system in eliciting immune reactions. For this reason, a reduction of the antigenic load in the graft by means of matching for HLA and most likely also of further (’minor’, H) transplantation antigens has a strong potential to reduce the risk of graft rejections.

## Methods/design

The trial is funded by the Deutsche Forschungsgemeinschaft (DFG). The study received appropriate ethics committees approval from the central ethics committee (University of Freiburg, 229/07) and the local ethics committees (University of Erlangen-Nuremberg, 4093-CH; University of Duisburg-Essen, 09–4134; University of Frankfurt, 234/09; Medical Association of Hamburg, MC-239/09; Medical Association of Saarland, 134/09; University of Kiel, B 255/09; Medical Association of Rheinland-Pfalz, 837.343.09 (6849); Ludwig-Maximilian-University of Munich, 294–09; University of Muenster, 2009-347-b-S; University of Wuerzburg, 31/10). Being registered at clinicaltrials.gov the unique identifying number of the FANCY trial is http://NCT00810472. Patient’s written consent was obtained prior to any study-specific procedures.

### Trial design

This is a prospective, controlled, randomised, double-blind multi-centre clinical trial with two parallel groups. The intervention is HLA matching for HLA-A, -B and -DRB1. Donors with less than 3 HLA mismatches are considered HLA matched. In the matching arm, the first donor exerting a 4/6 match (or “better“) is accepted within the first three months. A differential matching strategy on the basis of HLA-Matchmaker is additionally activated thereafter. The next available graft is assigned after 6 months. In the control arm, the next available donor is assigned. The matching arm is prioritised when a single graft could be allocated to more than one recipient.

### Objectives

The primary objective of the study is to demonstrate superiority of the proposed HLA matching strategy in comparison to random graft assignment with respect to the endpoint ‘time from keratoplasty to first endothelial graft rejection’ in penetrating keratoplasty. Secondary objectives of the study are: assessment of safety and tolerability; evaluation of time on waiting list and matching failures (failure to allocate a matching donor in the HLA matching arm within 6 months); retrospective analyses of the number and nature of mismatched antigens (HLA and minor transplantation antigens) in the patients with immune reactions; Retrospective analyses on the extent of antibody production against HLA mismatches in the rejecting patients.

### Setting

A multi-centre study design was chosen to account for patient recruitment with a reasonable time frame, for inclusion of a wider range of patients increasing the generalisability of the results and for the dissemination of findings when they become available. Trial sites are located in Erlangen, Essen, Frankfurt, Freiburg, Hamburg, Homburg/Saar, Kiel, Mainz, Munich, Muenster and Wuerzburg.

### Population

The target population of this trial is waiting for penetrating keratoplasty or lamellar endothelial keratoplasty. The majority of all indications for corneal transplantation are eligible for inclusion.

### Inclusion criteria

General inclusion criteria are: (1) patient’s written informed consent has been obtained, (2) 18 years or older at time of informed consent, (3) patient is awaiting either penetrating or endothelial lamellar keratoplasty. Indication-specific inclusion criteria are all corneal conditions that warrant either penetrating or lamellar endothelial keratoplasty.

### Exclusion criteria

General exclusion criteria are: (1) patient without legal capacity who is unable to understand the nature, significance and consequences of the study, (2) simultaneous participation in other interventional trials which could interfere with this trial and/or participation before the end of a required restriction period, (3) participation in a clinical trial within the last thirty days before the start of this study, (4) previous participation (randomisation) in this study, (5) known or persistent abuse of medication and/or drugs (such as alcohol), (6) persons who are in a relationship of dependence/employment with the sponsor or the investigator. An indication-specific exclusion criterion is predicted waiting time for a 4/6 HLA match greater than 6 months. The computation is performed by the trial software system on the basis of the patient's HLA phenotype and the HLA frequencies in the donor pool.

### Treatment arms

**Figure 1:**
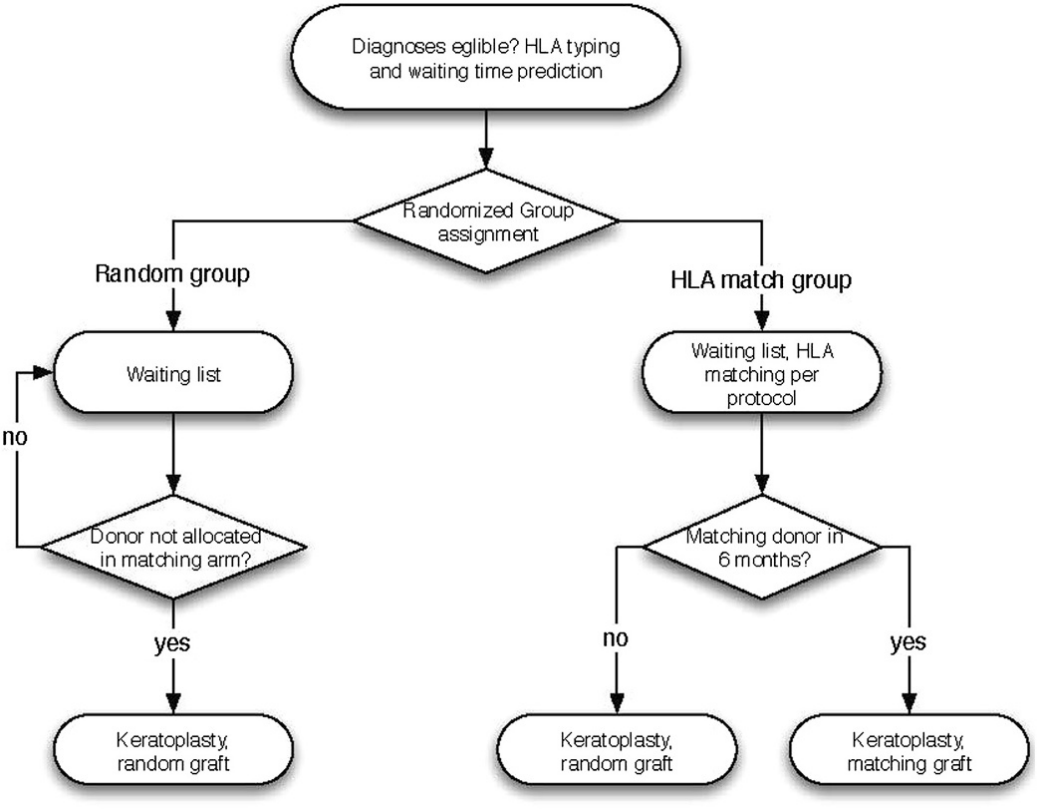
**Schematic of the allocation algorithm.** The matching arm is prioritised: the random arm is only assigned grafts that are not applicable to the matching arm at time of allocation.

All randomised trial patients on the waiting lists are matched against the donor-pool on a continuous basis. See Figure 1 for an overview of the underlying allocation algorithm. The matching arm is always prioritised: grafts are routed into the random arm when there is no match in the matching arm at time of allocation. Each patient in the matching arm is assigned a random graft after 6 months. The investigators are notified via email when a graft has been assigned to a trial patient. Whenever a keratoplasty is not possible on this basis, the investigator has to inform the trial coordinator as soon as possible (see the trial software manual). The waiting time limitation of 6 months in the matching arm is reset in this case.

### Visit schedule

We schedule a total of four follow up visits over a time span of two years post operatively (see Table 1). At each visit we ask for assessment of graft clarity and adverse events. We also demand careful recording of all concomitant medications.

**Table 1:** **Visit schedule**

Assessment	Screening	Preoperative visit	Keratoplasty	Visit 1	Visit 2	Visit 3	Close-out
Months/weeks after keratoplasty	Variable timeslot/within 6 month before keratoplasty	-1 week	= 0	4 (±3 weeks)	12 (±3 weeks)	18 (±3 weeks)	24 (±6 weeks)
Informed consent	x						
HLA typing	x						
Check eligibility	x						
Randomisation	x						
Demographic data	x	(x)*	(x)*				
Classification into high vs. low-risk patients	x	(x)*	(x)*				
Concomitant medications	x	(x)*		x	x	x	x
Endothelial cell density				x	x	x	x
Details on suturing		x	(x)*				
Details on trephination		x	(x)*				
Slit lamp evaluation of graft				x	x	x	x
Adverse events	x						
Post-OP complications in the study eye				x	x	x	x

*if data were not requested before.

The postoperative treatment and medical aftercare in corneal transplantation is recommended by the ’Sektion Kornea’ of the German Ophthalmologic Society (’Deutsche Ophthalmologische Gesellschaft’) in both study arms. Treatment of graft rejections is at the discretion of the investigator and may include topical, intracameral and systemic steroids.

### Outcome measures

The primary endpoint is the time interval from keratoplasty to the first graft rejection. Graft rejections are defined by either newly diagnosed keratic precipitates or rejection line in the donor endothelium, subepithelial infiltrates not explained by a preceding adenoviral conjunctivitis or newly diagnosed global graft edema that is otherwise not explained. The date of graft rejection is defined as the date on which treatment of the rejection is initiated. If no treatment was initiated, then the time of first mention of the rejection in the medical record is defined as date of the rejection.

Secondary endpoints are: (1) Number of HLA mismatches per patient in the matching arm, (2) number and nature of mismatched HLA/H transplantation antigens in the rejecting patients, (3) percentage of annual endothelial cell loss by means of exponential regression parameters [2, 3], (4) referring to substudy: ratio of HLA-antibody titers in blood samples after graft rejection to the respective titers prior to keratoplasty.

### Sample size

The sample size calculation is based on the primary endpoint ‘time to first endothelial graft rejection’. A rejection rate of 30% after 2 years is expected in the control group, and a reduction of events by 30% is considered to be clinically relevant, corresponding to a rejection rate of 21% after 2 years in the HLA matched group and a hazard ratio of 1.51. In order to detect this difference with a power of 80% at a 2-sided significance level of alpha = 5%, a total of 184 events is required. In order to account for the expected 10% match failures because of the intention-to-treat principle (see below), we randomly generated 1000 datasets, each with the proposed sample size of 620 and the expected matching effect of 30% after 3 years against a background of 30% immune reactions. After introducing 10% matching failures in each of these datasets, the mean matching effect dropped from 30% to 27%. These results in an assumed rejection rate of 21.9% in the HLA matched group (instead of 21% without any matching failures) and a hazard ratio of 1.44 (instead of 1.51). With an accrual period of 1.5 years for and a reference time of 1.5 years (almost all immune reactions have already occurred at this postoperative point in time), the power of the intention to treat analysis is 83%.

### Randomisation and blinding

Randomisation is performed, stratified by centre, in blocks of variable length. An equal distribution between treatment arms (ratio of 1:1) will be striven for. The randomisation list is generated by the Department of Biometry and Data Management of the Clinical Trial Unit, Medical Center – University of Freiburg.

The electronic version of the randomisation list was integrated in the trial software. Treatment assignment was thus performed via the trial software. In our study, this was the best possible way of blinding.

### Statistical analysis

The primary analysis of this clinical trial will be conducted according to the intention-to-treat (ITT) principle. This means that the patients will be analysed in the treatment arms to which they were randomised, irrespective of whether they refused or discontinued the treatment or whether other protocol violations are revealed. Patients not receiving keratoplasty cannot contribute any information to the analysis of treatment efficacy and will therefore be excluded from the ITT population.

The primary endpoint ‘time to first graft rejection’ will be analysed with a Cox proportional hazards model. The hazard ratio comparing control and treatment group will be estimated and tested within this model. The 2-sided 95% confidence interval will also be calculated. The Cox model will be stratified by study centre. We plan to include the recognised prognostic factors on immune reactions against the graft and overall graft survival in the model. Details will be fixed at the latest in a statistical analysis plan to be prepared prior to the analysis. The analysis of secondary endpoints ‘number of mismatches (HLA, H)’ will be performed descriptively, i.e. mean, median, range, standard deviation, and percentiles will be given. ‘Time on the waiting list’ will be estimated using the Kaplan Meier method separately in each treatment group, where patients not receiving keratoplasty are considered as censored observations.

### Study progress

The recruitment period was extended for another 12 months. During this period, a maximum of 280 patients were recruited per study centre, leading to a total of 654 patients.

### Quality assurance system

During the clinical trial, quality control and quality assurance will be ensured through monitoring, auditing and supervision by the authorities, if applicable.

An independent Data Monitoring Committee (DMC) was established. The function of the DMC is to monitor the course of the study and if necessary to give a recommendation to the study administration for discontinuation, modification or continuation of the study. The underlying principles for the DMC are ethical and safety aspects for the patients. It is the task of the DMC to examine, whether the conducting of the study is still ethically justifiable, whether security of the patients is ensured, and whether the process of the study is acceptable. For this, the DMC has to be informed about the adherence to the protocol, patient recruitment, and the observed adverse events. The DMC may also give a recommendation to extend the recruitment period and/or the maximum waiting period in the matching arm in case of an unexpectedly high percentage of matching failures.

## Discussion

FANCY was designed back in 2007. At that time, penetrating keratoplasty was the undisputed gold standard for corneal transplantation. Several trials on HLA matching in penetrating keratoplasty failed in the past for various reasons. In the UK, the CTFS II on DR matching was terminated prematurely because of under-recruitment [4]. The CCTS had issues with the typing quality [5]. Eventually, even HLA as the primary cause of rejection has been questioned on the basis of rodent experiments [6]. We carefully designed FANCY to work around all these issues. We abdicated almost all exclusion criteria, opted for molecular unambiguous two field HLA typing and included minor antigens into the analyses.

Lamellar techniques turned up at the horizon with descemet stripping automated endothelial keratoplasty (DSAEK) as the most prominent variant when we designed FANCY. At that time the immunologic risk in DSAEK and penetrating keratoplasty was considered quite comparable. However, during the course of the trial, Descemet Membrane Endothelial Keratoplasty (DMEK) became the new method of choice for surgical endothelial replacement therapy. It came to a big surprise that immune reactions turned out a subordinate problem with this novel method. This dramatically changed the landscape of keratoplasty. Nowadays, immune reactions are a significant clinical problem only in the high-risk keratoplasties. This is because immunologic endothelial graft failures after penetrating keratoplasty e.g. for keratoconus can also be treated with Descemet Membrane Endothelial Keratoplasty.

The primary goal of FANCY was to assess whether matching is feasable and effective for all patients within reasonable time. This is of subordinate relevance in 2014. If we would redesign FANCY in 2014, we would narrow down the inclusion criteria to include only the high risk patients and wait longer for a matching graft.

## Authors’ information

FANCY study group: PD Dr. Bjoern Bachmann (University Hospital Erlangen), Prof. Dr. Daniel Boehringer (Eye Center, Medical Center – University of Freiburg), Prof. Dr. Thomas Klink (University Hospital of Wuerzburg), Prof. Dr. Kohnen (University Hospital Frankfurt), PD Dr. Stephan Linke (University Medical Center Hamburg-Eppendorf), Dr. Katrin Lorenz (University Medical Center Mainz), Prof. Dr. Daniel Meller (University Hospital Essen), Prof. Dr. Elisabeth M. Messmer (Department of Ophthalmology, Ludwig-Maximilian-University, Munich), Dr. Bernhard Noelle (University Medical Center Schleswig-Holstein, Campus Kiel), Prof. Dr. Norbert Pfeiffer (University Medical Center Mainz), Prof. Dr. Berthold Seitz (Saarland University Medical Center), PD Dr. Constantin E. Uhlig (Department of Ophthalmology, University of Muenster Medical Center).

## Abbreviations

DMC: Data Monitoring Committee
HLA: Human leucocyte antigen.
